# Cost of healthcare for patients with migraine in five European countries: results from the International Burden of Migraine Study (IBMS)

**DOI:** 10.1007/s10194-012-0460-7

**Published:** 2012-05-29

**Authors:** L. M. Bloudek, M. Stokes, D. C. Buse, T. K. Wilcox, R. B. Lipton, P. J. Goadsby, S. F. Varon, A. M. Blumenfeld, Z. Katsarava, J. Pascual, M. Lanteri-Minet, P. Cortelli, P. Martelletti

**Affiliations:** 1 Allergan Inc., 2525 Dupont Drive, Irvine, CA 92612 USA; 2 United BioSource Corporation, Bethesda, MD USA; 3 Montefiore Headache Center and the Department of Neurology, Albert Einstein College of Medicine, Bronx, NY USA; 4 The Neurology Center, Encinitas, CA USA; 5 Headache Group, Department of Neurology, University of California, San Francisco, San Francisco, CA USA; 6 Department of Neurology, University of Essen, Essen, Germany; 7 Area of Clinical Neuroscience, Service of Neurology, University Hospital ‘‘Central de Asturias’’, Oviedo, Spain; 8 Département d’Evaluation et traitement de la Douleur Médecine palliative, Pôle Neurosciences Cliniques du CHU de Nice, Hôpital Cimiez, Nice Cedex, France; 9 IRCCS Institute of Neurological Sciences, Bologna, Italy; 10 Department of Neurological Sciences, Alma Mater Studiorum, University of Bologna, Bologna, Italy; 11 Department of Clinical and Molecular Medicine, Sapienza University of Rome, Regional Referral Headache Centre, Sant’Andrea Hospital, Rome, Italy

**Keywords:** Migraine, Chronic migraine, Episodic migraine, Cost, Resource utilization, United Kingdom, Spain, France, Italy, Germany

## Abstract

Migraine is a disabling neurological disease that affects 14.7 % of Europeans. Studies evaluating the economic impact of migraine are complex to conduct adequately and with time become outdated as healthcare systems evolve. This study sought to quantify and compare direct medical costs of chronic migraine (CM) and episodic migraine (EM) in five European countries. Cross-sectional data collected via a web-based survey were screened for migraine and classified as CM (≥15 headache days/month) or EM (<15 headache days/month), and included sociodemographics, resource use data and medication use. Unit cost data, gathered using publicly available sources, were analyzed for each type of service, stratified by migraine status. Univariate and multivariate log-normal regression models were used to examine the relationship between various factors and their impact on total healthcare costs. This economic analysis included data from respondents with migraine in the UK, France, Germany, Italy, and Spain. CM participants had higher level of disability and more prevalent psychiatric disorders compared to EM. CM participants had more provider visits, emergency department/hospital visits, and diagnostic tests; the medical costs were three times higher for CM than EM. Per patient annual costs were highest in the UK and Spain and lower in France and Germany. CM was associated with higher medical resource use and total costs compared to EM in all study countries, suggesting that treatments that reduce headache frequency could decrease the clinical and economic burden of migraine in Europe. Comparing patterns of care and outcomes among countries may facilitate the development of more cost-effective care, and bring greater recognition to patients affected by migraine.

## Introduction

Migraine is a complex and disabling neurological condition that produces substantial disability in nearly all facets of life of persons with migraine, including employment, household work, and social activities [1]. The World Health Organization (WHO) ranks migraine 19th among all causes of years lived with disability [2]. According to a recent review, 14.7 % of adults (8 % of men and 17 % of women) in Europe have migraine [3]. The cost of migraine in Europe is estimated at €27 billion annually due to the high prevalence of the disorder and substantial associated social, direct, and indirect costs [4, 5].

Migraine may be divided into two types based on headache frequency: episodic migraine (EM, <15 headache days per month) or chronic migraine (CM, ≥15 headache days per month). The International Classification of Headache Disorders Revised Criteria (ICHD-IIR) Appendix A1.5.1 defines chronic migraine as ≥15 headache days per month for at least 3 months, with ≥8 days per month fulfilling criteria for migraine without aura, in the absence of medication overuse and that cannot be attributed to another causative disorder [6]. The frequency of headache attacks in people with migraine may either increase or decrease over time. CM as a condition that often begins with episodic attacks which then increase in frequency over months or years to finally become a headache on more days than not or even a daily or almost daily mixture of tension-type headache and migraine [7]. Each year, approximately 2.5 % of persons with EM develop new-onset CM [8].

A recent prevalence estimate of CM in the US population in the American Migraine Prevalence and Prevention (AMPP) study among 162,756 individuals aged ≥12 years was found to be approximately 1 % (0.91 % overall, 1.29 % of females and 0.48 % of males) [9]. This study used criteria for EM and CM similar to those in the current study. Estimates of the worldwide prevalence of CM range from approximately 1.4–2.2 %, however, it the most common disorder seen in headache specialty practices and is often characterized as a condition that is both serious and difficult to treat [10, 11].

Both clinic and population-based studies have demonstrated that in comparison with those with EM, those with CM have greater headache-related disability, headache impact, worse socioeconomic status, worse health-related quality of life, higher rates of comorbid medical and psychiatric conditions, increased healthcare resource utilization, and higher direct and indirect costs [12–18].

Studies in Europe of those with chronic daily headache (experiencing ≤15 headache days per month), many of which may also meet the criteria as living with chronic migraine, have found significantly lower quality of life as measured by the SF-36, most notably the general health, vitality, and mental health concepts [19, 20].

Migraine prophylaxis is a major component of the management of migraine with the aim of reducing the frequency, duration, and severity of attacks as well as increasing the effect of acute treatment [21]. A number of different prophylactic therapies are either currently in use or being evaluated for the prevention of migraine. Economic burden of illness studies quantifying the differences in healthcare costs between CM and EM are particularly important in order to estimate the potential economic impact prophylactic agents may have either through reducing the transition from EM to CM or by facilitating CM remission.

In the United States and Canada, CM has been found to account for significantly higher healthcare costs compared to EM due to physician visits, emergency department visits, hospitalizations, and medications [18]. In addition to higher direct healthcare costs, the AMPP study has demonstrated a substantial indirect cost attributed to CM due to adverse effects on employment and productivity [17]. Although several studies have characterized the economic burden of migraine in general in Europe, to the best of our knowledge no study conducted to date has examined the impact of CM on healthcare costs [22–24]. Additionally, many of these studies were conducted using data collected prior to 1995 and results are likely not generalizable to today’s patients seeking migraine treatment [25]. The purpose of this work was to address these gaps using medical resource use data collected as part of the International Burden of Migraine Study (IBMS).

## Methods

### Data source and patient population

Data for the current economic analysis comes from a global, cross-sectional, web-based survey administered from February to April 2009. The core survey developed in English for the United States was subsequently adapted using country-specific validated translations of clinical and quality of life measures. A full description of the survey methods as well as the inclusion/exclusion criteria have been described elsewhere [26]. Individuals who had previously reported having experienced headaches or migraine were identified from panels maintained by Synovate Healthcare (Chicago, IL, USA). Study inclusion criteria included being at least 18 years of age, having an active e-mail address, the ability to read and understand the official language(s) in the participant’s country of residence, and a self-report of having headaches not associated with a cold, flu, head injury, or hangover in the past 3 months. Those agreeing to participate in the study provided consent by “opting in” using a web link provided in an e-mail invitation. A central ethics review board approved the study design and materials (Institutional Review Board Services, Ontario, Canada). Synovate provided participants with points, redeemable for modest cash rewards for their participation in the study.

Participants were selected via screening questions assessing the International Classification of Headache Disorders, 2nd Edition (ICHD-2) diagnostic criteria for migraine and were classified into chronic (≥15 headache days per month) or episodic (<15 headache days per month) migraine subgroups using headache frequency data. The ICHD-II criteria for CM were modified based on available data. Respondents were not assessed for meeting the ICHD-II criteria of ≥8 migraine days per month, thus meet criteria for chronic daily headache with a diagnosis of migraine disorder. Participants in the United Kingdom, Italy, Spain, France, and Germany were selected for inclusion into the study (*n* = 5,657).

### Sociodemographic and clinical study measures

Data on sociodemographic characteristics including age, gender, race, and education status were collected as part of the cross-sectional survey. Information related to comorbid conditions was collected via participants’ self-report of a physician diagnosis. Specific conditions were identified through response option endorsement to the question “Have you been told by a doctor or any other health professional that you have any of the following health problems?” Participants were categorized based on the conditions into five subgroups (psychiatric, pain, vascular risk factors, vascular disease events, and other). The survey also included a ten-point visual analog scale for the measurement of headache intensity. Intensity was categorized as follows: mild (≤4), moderate (5–7), severe (7 and 8), and very severe (9 and 10). Additionally, the survey measured disability using the Migraine Disability Assessment (MIDAS) questionnaire [27].

### Headache-related disability

The MIDAS questionnaire assesses headache-related disability and is the most frequently used disability instrument in migraine research and clinical practice [1, 28, 29]. It is a self-administered questionnaire consisting of five items that assess days of missed activity or substantially reduced activity due to headache in three domains—schoolwork/paid employment, household work or chores, and non-work (family, social, and leisure) activities. Responses to these items are summed for a total score, which can be categorized into one of four grades of headache-related disability: Grade I, little or no disability (score of 0–5); Grade II, mild disability (score of 6–10); Grade III, moderate disability (score of 11–20); and Grade IV, severe disability (score of ≥21).

MIDAS was originally developed and validated for use with a general headache sample. The standard MIDAS grading system groups patients with scores of 21 and above into a single category of “severe disability.” While this division works well for episodic migraine, a disproportionately large number of persons with chronic migraine fall into Grade IV. Accordingly, for this study, we subdivided the most severe category of Grade IV [30].

### Healthcare resource use data

Participants were asked to record the frequency of visits to various health care professionals occurring over the preceding 3 months for headache treatment or diagnostic evaluation. Participants could choose from various types of health care provider visits including primary care physician, neurologist, headache specialist, nurse practitioner, physician assistant, obstetrician/gynecologist, pain specialist, and psychologist, psychiatrist, or social worker. Choices for various categories of diagnostic testing included magnetic resonance imaging (MRI), computed tomography (CT), electroencephalogram (EEG), electrocardiogram (ECG), and blood tests. Data on the frequency with which specific headache-related procedures or devices including botulinum toxin type A injections, transcutaneous electronic nerve stimulator (TENS), and acupuncture were performed or prescribed during the preceding 3 months were collected. Participants were also asked to record the total number of nights spent in a hospital or clinic and the total number of visits to the emergency department (ED) for headache-related treatment in the preceding 3 months. Finally, study participants were provided with a list of medications commonly used as migraine acute and preventive pharmacotherapy unique to each participant’s country. Participants were then asked to identify any medications used in the preceding 4 weeks and to record the number of days of use for each medication. Data were gathered on acute and prophylactic medications and is listed by class in Table 4.

### Economic study data

Unit costs unique to each country were collected from publicly available sources and applied to health care resource use parameters (Table 1). Cost estimates were collected using a direct medical care perspective and standardized to €2010. In assigning costs, it was assumed that participants would receive care for a specific resource within the national or regional health system of his/her country of residence. Generally, the costs for each study participant were estimated by multiplying the frequency of use of each reported resource item by the unit cost for that item in the participant’s country of residence. If a specific procedure or medication was not reimbursed by the government, an assumption that the patient would either pay out of pocket or receive care in the private system was used, and thus not counted in this analysis. In Germany, physicians are reimbursed with quarterly lump sums for treating patients during each 3-month period or calendar year quarter regardless of the actual number of times patients are seen. Thus, the calculation of physician visit costs was modified to fit Germany’s reimbursement scheme.

**Table 1:** Unit cost estimates used for the United Kingdom, France, Italy, Spain, and Germany (in €2010)

Cost measure	Unit costs				
	UK	France	Italy	Spain	Germany
Primary care physician visit	€55 [34]	€23 [31]	€24 [35]	€32 [36]	€32 [32]
Neurologist/headache specialist visit	€165 [33]	€35 [31]	€24 [35]	€54 [36]	€32 [32]
Nurse practitioner/physician assistant visit	€13 [34]	€10 [31]	–	€13 [36]	–
OB/GYN visit	€114 [33]	€23 [31]	€24 [35]	€45 [36]	€28 [32]
Pain specialist visit	€126 [33]	€23 [31]	€24 [35]	€54 [36]	€24 [32]
Psychologist visit	€54 [34]	€35^a^	€20 [35]	€32 [36]	€19^a^
Psychiatrist visit	€250 [33]	€35 [31]	€20 [35]	€45 [36]	€19 [32]
Social worker visit	€47 [34]	€35^a^	–	€45^a^	€19^a^
ER or urgent care visit	€106 [33]	€25 [46]	€48^b^	€125 [46]	–
Inpatient hospital stay daily cost	€424 [33]	€402 [31]	€270 [43]	€485 [45]	€402 [44]
Magnetic resonance imaging (MRI)	€270 [33]	€69 [31]	€236 [35]	€166 [37]	€138 [32]
Computed tomography (CT)	€132 [33]	€25 [31]	€99 [35]	€199 [38]	€72 [32]
Electroencephalogram (EEG)	€135 [33]	€60 [31]	€24 [35]	€167 [36]	€38 [32]
Electrocardiogram (ECG)	€38 [33]	€14 [31]	€12 [35]	€7 [39]	€11 [32]
X-ray	€28 [33]	€24 [31]	€25 [35]	€37 [36]	€13 [32]
Blood tests	Various [33]	Various [31]	Various [35]	Various [38]	Various [32]
Botulinum toxin A injection					
Injection component	€165^c^	€35^c^	€24^c^	€54^c^	–
Botulinum toxin A drug component	€309 [51]	€450 [52]	€320 [50]	€438 [39]	€625 [49]
Transcutaneous nerve stimulator (TENS)	€53 [33]	€7 [31]	€11 [35]	€24 [39]	€7 [32]
Acupuncture	€45 [40]	€47 [41]	€18 [35]	€60 [42]	€32 [32]
Occipital nerve block	€247 [33]	€84 [31]	€53	€132 [39]	–
Medications	Various [47]	Various [31]	Various [50]	Various [48]	Various [49]

*NA* unit cost was not found for resource use item

^a^Cost estimate for specialist not found; assumed to be equal to psychiatrist visit

^b^Assumption, ER care = 2× cost of GP physician visit

^c^Assume injection cost is the same as a neurologist visit

Unit costs for most health care provider visits, diagnostic testing, and other procedures were estimated using the fee schedules representing payments made under the national systems for participants residing in France and Germany [31, 32]. In the UK, average costs published by the Department of Health were used [33, 34]. National price lists for Italy and Spain do not exist. For Italy, unit costs were estimated from the regional fee schedules of Lombardia [35]. Regional fee schedules from Galicia, País Vasco, Junta de Andalucía, and Comunitat Valenciana regions were used for Spain [36–39]. Unit costs for acupuncture were estimated using local websites for the UK, France, and Spain [40–42]. For Germany, the payment for acupuncture to treat chronic pain in the lower spine or knees under the public system was used as a proxy for migraine-related treatment. Estimates for nurse practitioner/physician assistant visits were not available for Italy and Germany; it was assumed that the costs for these providers were included in physician remuneration.

Hospital costs were estimated using costs based on the public system reference cost groupers for headache or migraine-related hospital care available in each country [31, 33, 43–45]. Reimbursements paid to hospitals are based on a flat rate per admission which is set according to diagnosis-related groups. In assigning costs, the most conservative or least expensive grouper code was used. ED visits were estimated using public health system data for the UK, France and Spain [33, 36, 46]. ED visit cost estimates could not be identified for Italy and Germany. For Italy, an ED visit was assumed to be twice the cost of a primary care physician visit. As urgent care in Germany is routinely managed by general practitioners, ED visits were assumed to be included in the remuneration of primary care physicians.

Medication cost estimates were obtained from the national formularies of each study country with the exception of Italy [31, 47–49]. For Italy, medication costs were identified from a private site for health care professionals [50]. Since data on medication dose were not collected as part of the internet survey, all medication costs were estimated using daily dose assumptions based on the expert clinical opinion of the study authors who are also physicians. If more than one dose was plausible for a given medication, the midpoint of the plausible range was chosen. For example, for countries in which the price per vial of botulinum toxin type A was available, an average dose of 150 units was assumed in estimating costs since doses in the plausible range of 100–200 units likely would have been prescribed. In the UK and France, botulinum toxin type A unit costs could not be identified using the NHS reference data or the national fee schedules, respectively. Costs were estimated using information obtained from on-line sources [51, 52]. For all countries, we assumed that the cost associated with the administration of botulinum toxin A is equal to one neurologist/headache specialist visit.

### Data analyses

Baseline sociodemographic and clinical characteristics including age, gender, race, education, headache-related disability (as measured by MIDAS score), headache intensity, and medical and psychiatric comorbidities were assessed descriptively for each country. Within countries, comparisons between CM and EM groups were made using two-sided Pearson Chi-square for categorical measures and *t* test statistics for continuous measures. Health care use measures were also summarized using descriptive statistics. Because of the relatively low frequency, some health care resource use items (e.g., hospitalizations), Fisher’s exact test was used for group comparisons. Initial data analyses revealed that two participants had very high costs. Upon examination of these patients’ resource use profiles, they were excluded from the analysis because of implausibly high values. Statistically significant differences were evaluated at an α = 0.05.

Healthcare costs were analyzed separately for each resource use category and stratified by migraine status for each of the study countries. A minority of study participants had missing data due to inability to recall medication name or frequency. Health care costs are presented over 3 months as well as annually. Costs were annualized by multiplying the 3-month average healthcare cost by 4. Total costs were estimated by summing each individual category.

Analyses of the determinates of costs related to migraine headaches with a particular focus on the impact of headache frequency (CM vs. EM) were conducted using multivariate methods. Gamma regression models with log-link function were fitted separately for each study country to explore the relationship between various factors hypothesized to have an impact on total healthcare costs over 3 months (CM status, MIDAS disability scores, headache intensity, and comorbidities). Associations in these models were measured in terms of expected differences in means of the log of total costs with 95 % confidence intervals. We assessed each determinant separately (in models that included age, gender, and education) and then jointly in a multivariate model. MIDAS disability scores were excluded from multivariate models due to expected collinearity with headache frequency.

## Results

### Sociodemographic and clinical characteristics of participants

Sociodemographic and clinical characteristics of the study participants are presented in Table 2 by study country and migraine status. Overall, study groups were comparable with respect to nearly all sociodemographic parameters studied. However, with respect to clinical parameters there were notable differences between groups. For all countries, CM participants reported higher levels of headache-related disability compared to persons with EM (*P* < 0.001, all comparisons). The proportion of CM participants with severe headache-related disability (MIDAS Grade IV-A or IV-B) ranged from 71.4 % in Spain to 90.4 % in Germany, whereas less than one-third of EM participants were classified as Grade IV-A or IV-B (UK 24.0 %, France 20.4 %, Germany 34.6 %, Italy 33.5 %, and Spain 23.7 %). Across all countries, psychiatric disorders were more prevalent among CM in comparison to EM (*P* < 0.05, all comparisons). Overall, almost one-half (45.5 %) of CM participants reported having a psychiatric disorder compared to approximately one-third (27.9 %) of EM participants. In all countries with the exception of the UK, a greater proportion of participants reported having a comorbidity related to pain compared to EM (*P* < 0.05 for France and Italy and *P* < 0.001 for Germany and Spain). In France, approximately one-third (33.3 %) of CM participants reported having a vascular disease risk factor versus one-quarter (24.6 %) of EM participants (*P* < 0.05).

**Table 2:** Characteristics of participants with chronic (CM) and episodic migraine (EM) in the UK, France, Germany, Italy, and Spain

Characteristic^a^	UK		France		Germany		Italy		Spain	
	CM (*n* = 57)	EM (*n* = 1,013)	CM (*n* = 57)	EM (*n* = 1404)	CM (*n* = 52)	EM (*n* = 1397)	CM (*n* = 55)	EM (*n* = 921)	CM (*n* = 56)	EM (*n* = 645)
Age, mean (SD)	42.9 (12.7)	44.3 (11.2)	40.8 (11.4)	37.8 (10.1)*	37.9 (11.9)	38.0 (10.8)	34.5 (10.9)	37.1 (10.0)	36.2 (10.8)	35.1 (9.0)
Female (%)	82.50	84.80	87.70	89.60	78.80	81.80	90.90	80.60	85.70	79.20
Race/ethnicity (%)										
White/Caucasian	98.20	94.70	93.00	95.70	92.30	98.50*	96.40	93.70	62.50	75.30
Black	1.80	1.30	0	1.40	0	0	0	0.30	1.80	0.30
Asian	0	1.90	0	0.20	0	0.40	0	0.30	0	0.30
Hispanic or Latino/Latin American	0	0.30	1.80	0.60	1.90	0.20	3.60	4.20	33.90	22.60
Other/prefer not to answer	0	1.90	5.30	2.10	5.80	0.90	0	1.70	1.80	1.40
Education (%)										
Less than a high school diploma	0	2.80	22.80	15.20	53.80	48.80	12.70	7.90	5.40	2.80
High school graduate	28.10	26.80	21.10	19.40	13.50	19.30	43.60	43.30	16.10	18.40
Some college/Associates degree	43.90	37.70	19.30	19.00	15.40	11.00	25.50	21.00	37.50	31.20
College or professional degree	22.80	26.40	28.10	41.50	15.40	18.50	18.20	27.00	33.90	45.60
Other/prefer not to answer	5.30	6.40	8.80	4.90	1.90	2.40	0	0.80	7.10	2.00
MIDAS (%)										
Grade I, little disability (0–5)	5.30	27.7**	3.50	35.30**	1.90	20.30**	5.50	22.6**	12.50	32.90**
Grade II, mild disability (6–10)	1.80	21.80	7.00	22.50	1.90	19.30	1.80	19.30	3.60	17.40
Grade III, moderate disability (11–20)	5.30	26.50	10.50	21.70	5.80	25.80	7.30	24.50	12.50	26.00
Grade IV-A, severe disability (21–40)	14.00	16.70	15.80	14.80	13.50	24.10	9.10	21.60	14.30	16.90
Grade IV-B, very severe disability (41–270)	73.70	7.30	63.20	5.60	76.90	10.50	76.40	11.90	57.10	6.80
Headache intensity (%)										
Mild (0–3)	0	2.20	0	2.90	0	2.40	1.80	4.20*	1.80	5.90*
Moderate (4–6)	5.30	13.40	17.50	21.70	11.50	13.00	0	17.00	3.60	18.10
Severe (7–9)	57.90	51.30	56.10	58.20	48.10	57.30	60.00	55.60	55.40	55.20
Very severe (9–10)	36.80	33.10	26.30	17.20	40.40	27.30	38.20	23.10	39.30	20.80
Comorbidity groups^b^ (%)										
Pain-related	29.80	21.70	24.60	12.10*	40.40	17.00**	21.80	11.20*	37.50	13.5**
Vascular disease risk factors	38.60	29.10	33.30	24.0*	36.50	27.90	32.70	28.30	32.10	27.60
Vascular disease events	7.00	3.50	0	1.30	5.80	3.90	5.50	3.30	8.90	2.20*
Psychiatric disorders	49.10	31.30*	38.60	26.40*	42.30	22.50*	50.90	33.00*	46.40	30.20*
Other conditions	33.30	32.80	47.40	33.80*	46.20	30.30*	54.50	42.60	44.60	39.40

*SD* standard deviation*, n* number of participants

* Comparison significant at *P* < 0.05

** Comparison significant at *P* < 0.001

^a^Group comparisons were made using two-sided Pearson Chi-square for categorical measures and *t* test statistics for continuous measures

^b^Comorbidity was also assessed by having participants record whether or not they had ever been told by a physician that they have conditions related to pain, vascular disease, vascular events, or psychiatric disorders

### Healthcare resource use related to migraine

**Fig. 1:**
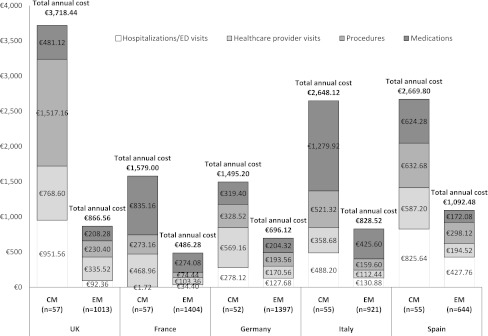
Mean total annual costs per patient by migraine group

Table 3 presents data on headache-related medical resources (excluding medications) used by the study participants in the past 3 months and Fig. 1 presents the annualized mean total healthcare costs per patient by migraine group and country. Overall, the most common services utilized by migraine participants were healthcare provider visits, diagnostic testing, and blood tests. The utilization of these services was similar across countries. Over half (54.5 %) of European CM participants reported visiting a primary care physician for headache over the previous 3 months, in contrast to only one-third (29.8 %) of EM (*P* < 0.05, for each comparison within countries). Differences in the extent in which CM and EM participants utilized neurologist/headache specialist visits were also striking with nearly one-third (30.7 %) and 9.7 % of CM and EM, respectively, reporting having visited a neurologist or headache specialist (*P* < 0.001). CM participants also utilized diagnostic testing to a greater extent compared with EM in the UK (CM 14.0 %; EM 4.6 %, *P* < 0.05), France (CM 28.1 %; EM 10.4 %, *P* < 0.001), and Spain (CM 21.4 %; EM 9.5 %, *P* < 0.05). Blood tests were also utilized to a much greater extent among CM versus EM participants in the UK (12.3 vs. 4.0 %, *P* < 0.05), France (17.5 vs. 6.8 %, *P* < 0.05), Germany (21.2 vs. 8.6 %, *P* < 0.05), and Spain (21.4 vs. 6.4 %, *P* < 0.001).

**Table 3:** Health care resource use during preceding 3 months among participants with chronic (CM) and episodic migraine (EM) in the UK, France, Germany, Italy, and Spain

Study measure^a^	UK (*N* = 1,070)		France (*N* = 1,461)		Germany (*N* = 1,449)		Italy (*N* = 976)		Spain (*N* = 699)	
	CM (*n* = 57)	EM (*n* = 1013)	CM (*n* = 57)	EM (*n* = 1,404)	CM (*n* = 52)	EM (*n* = 1397)	CM (*n* = 55)	EM (*n* = 921)	CM (*n* = 55)	EM (*n* = 644)
Primary care physician visits (%)	56.10	28.3**	59.60	36.5**	48.10	29.1*	52.70	25.5**	54.50	25.30**
Mean (SD)^b^	2.57 (1.55)	2.54 (3.43)	5.10 (8.91)	1.97 (1.64)	5.82 (5.78)	2.65 (2.28)	4.14 (3.20)	2.81 (3.33)	4.82 (4.36)	2.55 (2.35)
Min–max	1–6	1–35	1–50	1–20	1–25	1–20	1–10	1–30	1–20	1–20
Neurologist/headache specialist visits (%)	28.10	6.0**	17.50	5.1**	34.60	12.7**	40.00	14.7**	32.70	11.50**
Mean (SD)	1.53 (1.06)	1.73 (1.10)	1.80 (1.03)	1.46 (0.76)	3.13 (2.58)	1.92 (1.55)	2.18 (1.76)	1.83 (1.80)	1.53 (0.87)	1.50 (1.06)
Min–max	1–5	1–5	1–4	1–5	1–10	1–10	1–8	1–12	1–4	1–6
Nurse practitioner/physician assistant visits (%)	3.50	3.30	1.80	0.40	1.90	1.30	1.80	1.50	7.30	4.20
Mean (SD)	3.00 (2.83)	3.28 (5.24)	2.00^d^	17.67 (35.49)	5.00^d^	2.47 (1.66)	1.00^d^	2.31 (1.38)	2.75 (2.22)	2.35 (1.50)
Min–max	1–5	1–29	2–2	1–90	5–5	1–6	1–1	1–5	1–6	1–8
Other specialist visits^c^ (%)	14.00	6.70	26.30	9.9**	26.90	12.5*	29.10	9.1**	23.60	12.70*
Mean (SD)	2.14 (1.46)	3.83 (5.89)	5.17 (8.95)	1.94 (1.99)	4.15 (3.29)	3.58 (4.12)	3.57 (2.65)	2.39 (2.43)	3.23 (2.59)	2.75 (2.89)
Min–max	1–5	1–30	1–31	1–18	1–10	1–21	1–8	1–12	1–9	1–16
Emergency room visits (%)	12.30	3.5*	1.80	2.10	3.80	3.90	5.50	6.40	27.30	15.50*
Mean (SD)	4.14 (5.05)	1.86 (2.17)	1.00^d^	1.50 (0.92)	2.00 (1.41)	1.83 (1.78)	4.50 (2.12)	1.72 (1.50)	2.75 (2.01)	2.32 (2.62)
Min–max	1–15	1–10	1–1	1–5	1–3	1–10	3–6	1–10	1–7	1–17
Hospitalizations (%)	8.80	1.5*	0	1.40	3.80	1.60	3.60	2.40	3.60	3.60
Mean LOS (SD)	4.40 (6.27)	2.13 (1.88)	–	1.37 (1.12)	3.50 (4.95)	3.14 (3.38)	10.00 (7.07)	3.14 (3.03)	6.00 (1.41)	3.13 (6.26)
Min–max	0–15	0–8	–	0–5	0–7	0–10	5–15	0–8	5–7	0–30
Diagnostic testing^e^ (%)	14.00	4.6*	28.10	10.4**	23.10	12.90	16.40	10.20	20.00	9.30*
Mean (SD)	3.13 (2.90)	3.30 (7.37)	2.53 (1.60)	1.54 (0.96)	3.00 (3.16)	2.87 (2.25)	5.00 (4.39)	2.61 (1.67)	2.64 (1.75)	2.81 (4.08)
Min–max	1–10	1–48	1–6	1–6	1–11	1–20	1–15	1–9	1–6	1–30
Blood tests (%)	12.30	4.0*	17.50	6.8*	21.20	8.6*	12.70	9.10	20.00	6.40*
Mean (SD)	3.57 (3.82)	2.22 (1.90)	2.11 (1.27)	1.24 (0.48)	2.11 (1.05)	1.48 (0.81)	2.71 (2.06)	1.57 (1.03)	2.09 (2.70)	1.55 (0.83)
Min–max	1–12	1–9	1–5	1–3	1–4	1–4	1–6	1–5	1–10	1–4
Botulinum toxin A injections (%)	3.50	0.60	0	0.10	0	1.10	3.60	0.70	1.80	1.90
Mean (SD)	3.00^d^	2.00 (2.00)	–	2.00^d^	–	2.08 (1.50)	4.00 (1.41)	2.00 (1.41)	1.00^d^	1.67 (1.23)
Min–max	3–3	1–5	–	2–2	–	1–5	3–5	1–4	1–1	1–5
Transcutaneous nerve stimulator procedures (%)	5.30	3.80	0	1.10	3.80	1.60	1.80	2.70	10.90	1.60**
Mean (SD)	20.50 (27.58)	6.87 (6.43)	–	14.70 (12.66)	20.00^d^	7.69 (10.25)	5.00^d^	8.71 (9.13)	3.75 (4.19)	9.25 (10.46)
Min–max	1–40	1–30	–	2–40	20–20	1–40	5–5	1–30	1–10	1–30
Acupuncture (%)	7.00	3.50	8.80	3.80	11.50	6.90	5.50	4.10	12.70	3.70*
Mean (SD)	1.00 (0.00)	3.00 (2.36)	7.00 (5.61)	3.18 (2.72)	10.80 (4.27)	5.33 (4.11)	3.67 (3.06)	4.30 (3.76)	7.60 (5.32)	5.00 (4.86)
Min–max	1–1	1–8	1–15	1–15	5–15	1–15	1–7	1–15	2–15	1–15
Occipital nerve block procedures (%)	12.30	0.9**	0	0.40	0	2.10	1.80	1.40	1.80	2.20
Mean (SD)	6.67 (6.62)	5.50 (3.46)	–	1.50 (0.71)	–	2.45 (1.47)	3.00^d^	1.88 (0.83)	1.00^d^	4.86 (4.74)
Min–max	1–15	1–12	–	1–2	–	1–6	3–3	1–3	1–1	1–12

–, mean (SD) could not be calculated because 0 patients received a medical resource in the category

*SD* standard deviation*, n* number of patients

* Comparison significant at *P* < 0.05

** Comparison significant at *P* < 0.001

^a^Group comparisons were made using the Fisher’s exact test

^b^Mean number of events of those reporting 1 or more

^c^Includes psychologist, psychiatrist, social worker, pain specialist, and OB/GYN visits

^d^SD not calculated because data were reported by only one respondent

^e^Includes magnetic resonance imaging, computed tomography, electroencephalogram, and electrocardiogram tests

Overall, the proportion of participants reporting an ED visit was much higher in Spain (16 %) compared to the UK (4 %) France (2 %), Germany (4 %), and Italy (6 %). CM participants were more likely to report having an ED visit compared to EM in the UK (12.3 and 3.5 %, respectively, *P* < 0.05) and Spain (28.6 and 15.5 %, respectively, *P* < 0.05). The proportion reporting a hospitalization for migraine treatment was also higher in Spain (3.6 %) compared to the UK (1.9 %), France (1.3 %), Germany (1.7 %), and Italy (2.5 %). However, only in the UK was the use of hospital services significantly higher for CM versus EM participants (CM 8.8 %; EM 1.5 %, *P* < 0.05) (Table 3).

Table 4 displays acute and prophylactic medication use data by medication class. The frequency of over the counter or prescription medication use varied across study countries. Medication use was highest in the UK (75.4 %) and France (72.5 %), followed by Italy (65.1 %), Spain (64.1 %) and Germany (49.0 %). Medication use among CM participants was significantly higher compared to EM in France (CM 87.7 %; EM 71.9 %, *P* < 0.05), Italy (CM 83.6 %; EM 64.0 %, *P* < 0.05), and Spain (CM 91.1 %; EM 61.9 %, *P* < 0.05) but not in the UK or Germany. CM participants used acute medications to a greater extent compared to EM in Italy (CM 56.4 %; EM 35.5 %, *P* < 0.05) and Spain (CM 87.3 %; EM 59.2 %, *P* < 0.05). Use of prophylactic medications was significantly higher in CM versus EM participants in Spain only (CM 23.6 %; EM 7.5 %, *P* < 0.05) (Table 5).

**Table 4:** Medication use in the past 4 weeks for participants with chronic (CM) and episodic migraine (EM) in the UK, France, Germany, Italy, and Spain

Medication class	UK (*N* = 1,070)		France (*N* = 1,461)		Germany (*N* = 1,449)		Italy (*N* = 976)		Spain (*N* = 699)^a^	
	CM (*n* = 57)	EM (*n* = 1,013)	CM (*n* = 57)	EM (*n* = 1,404)	CM (*n* = 52)	EM (*n* = 1,397)	CM (*n* = 55)	EM (*n* = 921)	CM (*n* = 55)	EM (*n* = 644)
Over the counter or other prescription medication used (% yes)	66.70	75.90	87.70	71.90	44.20	49.20	83.60	64.00	90.90	61.80
Acute Medication Use (% yes)	57.90	63.70	52.60	47.60	34.60	45.80	56.40	35.50	87.30	59.20
Combination with Opioids (% yes)	45.60	49.50	31.60	34.30	13.50	14.00	30.90	17.20	49.10	23.00
Mean days (SD)	20.43 (8.70)	7.08 (6.95)	15.55 (10.02)	5.80 (5.38)	12.33 (9.46)	5.72 (4.97)	13.00 (10.67)	4.83 (4.58)	15.84 (9.07)	4.52 (4.55)
Combination without Opioids (% yes)	7.00	10.00	N/A	N/A	5.80	6.90	12.70	6.40	10.90	5.00
Mean days (SD)	16.25 (9.60)	5.33 (5.00)	N/A	N/A	10.00 (0.00)	5.15 (4.59)	15.00 (13.91)	5.49 (5.45)	21.67 (5.69)	5.70 (6.82)
Ergotamines (% yes)	N/A	N/A	3.50	1.90	N/A	N/A	14.50	3.40	20.00	9.80
Mean days (SD)	N/A	N/A	17.50 (14.85)	18.26 (11.83)	N/A	N/A	8.60 (6.99)	5.00 (4.58)	15.43 (9.76)	4.02 (3.50)
NSAIDS (% yes)	N/A	N/A	N/A	N/A	0.00	0.40	10.90	7.90	78.20	49.40
Mean days (SD)	N/A	N/A	N/A	N/A	–	7.50 (3.32)	12.00 (5.29)	5.03 (4.84)	17.72 (9.62)	6.08 (5.55)
Simple analgesics (% yes)	N/A	N/A	N/A	N/A	26.90	37.10	N/A	N/A	16.40	12.00
Mean days (SD)	N/A	N/A	N/A	N/A	16.80 (10.75)	6.68 (5.94)	N/A	N/A	11.00 (9.56)	3.43 (3.65)
Triptans (% yes)	28.10	24.50	26.30	20.80	5.80	14.50	21.80	15.10	29.10	16.50
Mean days (SD)	12.18 (7.19)	5.22 (5.28)	11.67 (9.53)	4.31 (4.79)	14.50 (16.26)	4.85 (4.59)	10.70 (9.36)	4.80 (5.14)	11.93 (8.87)	3.82 (4.32)
Preventive (% yes)	31.60	27.60	19.30	9.00	9.60	10.20	3.60	4.10	23.60	7.50
Antidepressants (% yes)	22.80	17.60	1.80	2.00	7.70	7.20	N/A	N/A	7.30	2.50
Mean days (SD)	28.00 (0.00)	23.16 (8.97)	28.00^b^	13.96 (11.91)	12.50 (10.61)	13.78 (10.72)	N/A	N/A	24.50 (7.00)	18.14 (11.88)
Antiepileptics (% yes)	8.80	5.00	5.30	2.80	0.00	1.00	3.60	1.50	9.10	3.00
Mean days (SD)	28.00 (0.00)	21.58 (10.27)	28.00 (0.00)	17.70 (11.33)	–	15.82 (12.01)	2.00^b^	4.57 (4.13)	20.60 (8.59)	20.53 (10.29)
Beta blockers and calcium channel blockers (% yes)	10.50	5.70	10.50	3.80	1.90	3.10	0.00	2.00	12.70	3.10
Mean days (SD)	28.00 (0.00)	23.19 (9.14)	23.40 (10.29)	22.24 (10.44)	10.00^b^	21.31 (10.88)	–	13.14 (11.20)	18.80 (12.77)	16.95 (12.32)
Serotonergic (% yes)	7.00	5.10	3.50	1.90	0.00	0.10	0.00	1.60	N/A	N/A
Mean days (SD)	21.50 (13.00)	23.43 (9.21)	21.00 (9.90)	15.23 (11.96)	–	8.00^b^	–	8.73 (9.95)	N/A	N/A
Other drugs (% yes)	38.60	32.30	63.20	37.60	19.20	16.80	50.90	36.20	16.40	14.60
Mean days (SD)	22.29 (7.63)	7.92 (8.39)	19.50 (7.36)	6.85 (6.66)	17.75 (9.82)	6.73 (7.04)	16.45 (7.89)	7.02 (7.08)	19.71 (10.50)	6.84 (6.42)
Do not know the medication name (% yes)	0.00	1.10	3.50	2.90	1.90	0.50	3.60	4.20	0.00	0.30
Mean days (SD)	–	7.89 (11.43)	28.00^b^	6.32 (5.50)	–	7.25 (4.50)	21.50 (9.19)	8.04 (8.80)	–	2.00^b^
Total medications										
Mean (SD)	2.68 (1.16)	2.18 (1.28)	1.72 (0.88)	1.56 (0.87)	2.04 (1.02)	2.23 (1.31)	2.07 (2.11)	1.63 (1.54)	3.36 (1.79)	2.62 (1.58)
Minimum–maximum	1–6	1–22	1–4	1–10	1–4	1–10	1–15	1–22	1–8	1–13
Median	3	2	2	1	2	2	2	1	3	2
Number of classes of medications used^c^										
Mean (SD)	2.53 (1.11)	1.99 (0.96)	1.70 (0.84)	1.50 (0.71)	1.87 (0.92)	2.06 (1.07)	1.78 (0.89)	1.49 (0.86)	2.74 (1.41)	2.25 (1.14)
Minimum–maximum	1–5	1–8	1–4	1–6	1–4	1–6	1–5	1–8	1–6	1–7
Median	3	2	2	1	2	2	2	1	3	2

Medication classes may include one or more medications of that type. For participants using more than one medication type within a class, medication days were calculated using the maximum duration of all drugs in a particular class

–, mean (SD) could not be calculated because 0 patients received medications in the therapeutic class or did not report number of days of use

*SD* standard deviation, *n* number of patients, *N/A* no medications in the therapeutic class were included in the survey for that country

^a^Two patients were excluded because they had extreme values for certain RU parameters (One patient with 45 ER visits and 20 hospitalizations and one patient with 25 X-ray tests

^b^SD not calculated because data were reported by only one respondent

^c^Includes participants who had used an OTC or prescription medication to treat headache. Other and do not know the medication name were counted as a single medication class

**Table 5:** Health care costs (in €2010) over 3 months for participants with chronic (CM) and episodic migraine (EM) in the UK, France, Germany, Italy, and Spain

Study measurement	UK (*N* = 1,070)		France (*N* = 1,461)		Germany (*N* = 1,449)		Italy (*N* = 976)		Spain (*N* = 699)^a^	
	CM (*n* = 57)	EM (*n* = 1,013)	CM (*n* = 57)	EM (*n* = 1,404)	CM (*n* = 52)	EM (*n* = 1,397)	CM (*n* = 55)	EM (*n* = 921)	CM (*n* = 55)	EM (*n* = 644)
Primary care physician visits										
Mean (SD)	79.67 (94.01)	39.82 (116.6)	64.57 (156.7)	16.71 (31.31)	82.67 (147.1)	24.36 (53.61)	47.72 (66.02)	16.94 (47.88)	81.55 (123.9)	20.79 (50.89)
Min–max	0–332	0–1,936	0–1,159	0–464	0–788	0–630	0–235	0–706	0–636	0–636
Median	55	0	23	0	0	0	24	0	32	0
Neurologist/headache specialist visit										
Mean (SD)	71.38 (144.7)	17.15 (79.96)	11.03 (28.12)	2.61 (12.65)	32.75 (63.62)	7.70 (26.24)	20.54 (36.24)	6.32 (21.99)	27.04 (46.75)	9.32 (31.91)
Min–max	0–824	0–824	0–140	0–175	0–315	0–315	0–188	0–282	0–215	0–322
Median	0	0	0	0	0	0	0	0	0	0
Nurse practitioner/physician assistant visits										
Mean (SD)	1.36 (8.71)	1.38 (14.08)	0.35 (2.67)	0.76 (24.28)	0.00 (0.00)	0.00 (0.00)	0.00 (0.00)*	0.00 (0.00)*	2.67 (11.88)	1.32 (7.31)
Min–max	0–65	0–375	0–20	0–906	0–0	0–0	0–0	0–0	0–80	0–107
Median	0	0	0	0	0	0	0	0	0	0
Other specialist visits										
Mean (SD)	39.74 (122.5)	25.53 (181.6)	41.29 (141.2)	5.76 (26.64)	26.87 (57.08)	10.58 (42.28)	21.41 (43.54)	4.85 (21.53)	35.54 (86.33)	17.20 (71.89)
Min–max	0–676	0–3,400	0–899	0–594	0–244	0–513	0–171	0–273	0–416	0–1,056
Median	0	0	0	0	0	0	0	0	0	0
Emergency room visits										
Mean (SD)	53.89 (227.5)	7.09 (53.63)	0.43 (3.28)	0.79 (6.23)	0.00 (0.00)	0.00 (0.00)	9.25 (43.51)	5.19 (26.39)	93.61 (191.1)	45.64 (162.1)
Min–max	0–1,589	0–1,059	0–25	0–124	0–0	0–0	0–282	0–471	0–872	0–2,117
Median	0	0	0	0	0	0	0	0	0	0
Hospitalizations										
Mean (SD)	184.0 (939.2)	16.00 (171.0)	0.00 (0.00)	7.81 (83.91)	69.53 (448.1)	31.92 (333.2)	112.8 (630.9)	27.53 (217.6)	112.8 (591.9)	61.30 (649.0)
Min–max	0–6,570	0–4,133	0–0	0–2,035	0–3,214	0–7,231	0–4,316	0–2,698	0–3,527	0–14,706
Median	0	0	0	0	0	0	0	0	0	0
Diagnostic testing^b^										
Mean (SD)	81.69 (290.1)	19.97 (195.3)	26.43 (52.83)	6.78 (25.07)	38.15 (95.96)	20.62 (75.81)	64.02 (192.4)	24.07 (85.23)	70.90 (160.7)	29.83 (167.6)
Min–max	0–2,007	0–5,655	0–223	0–352	0–500	0–1,285	0–1,201	0–596	0–616	0–3,651
Median	0	0	0	0	0	0	0	0	0	0
Blood tests										
Mean (SD)	5.16 (20.27)	1.07 (6.69)	13.00 (33.57)	3.11 (12.32)	3.37 (7.41)	1.01 (3.79)	8.94 (29.55)	3.72 (14.09)	10.50 (36.05)	2.56 (11.08)
Min–max	0–141	0–106	0–182	0–109	0–32	0–32	0–155	0–129	0–251	0–100
Median	0	0	0	0	0	0	0	0	0	0
Botulinum toxin injections										
Mean (SD)	43.22 (231.4)	5.80 (91.23)	0.00 (0.00)	1.38 (36.54)	0.00 (0.00)	13.93 (159.4)	49.93 (267.7)	4.97 (67.94)	8.84 (65.57)	15.10 (134.7)
Min–max	0–1,421	0–2,369	0–0	0–969	0–0	0–3,124	0–1,716	0–1,373	0–486	0–2,431
Median	0	0	0	0	0	0	0	0	0	0
Transcutaneous nerve stimulator procedures (TENS)										
Mean (SD)	45.11 (284.6)	13.93 (91.15)	0.00 (0.00)	1.10 (12.25)	3.63 (19.87)	0.83 (9.62)	0.96 (7.13)	2.50 (20.64)	12.19 (43.57)	3.17 (35.90)
Min–max	0–2,119	0–1,589	0–0	0–263	0–133	0–266	0–53	0–317	0–232	0–697
Median	0	0	0	0	0	0	0	0	0	0
Acupuncture										
Mean (SD)	4.61 (19.50)	4.63 (31.04)	28.86 (117.4)	5.79 (37.68)	36.98 (112.3)	12.00 (54.95)	3.59 (18.43)	3.20 (19.95)	53.38 (166.8)	11.35 (76.39)
Min–max	0–128	0–358	0–705	0–705	0–483	0–483	0–126	0–269	0–898	0–898
Median	0	0	0	0	0	0	0	0	0	0
Occipital nerve block										
Mean (SD)	199.5 (727.3)	12.20 (147.3)	0.00 (0.00)	0.45 (7.64)	0.00 (0.00)	0.00 (0.00)	2.89 (21.46)	1.44 (12.65)	2.36 (17.54)	12.52 (103.3)
Min–max	0–3,708	0–2,966	0–0	0–167	0–0	0–0	0–159	0–159	0–130	0–1,561
Median	0	0	0	0	0	0	0	0	0	0
Prophylactic medications										
Mean (SD)	40.50 (93.48)	17.73 (54.80)	7.08 (19.41)	3.27 (14.88)	4.07 (14.30)	4.90 (25.86)	0.48 (2.68)	0.91 (5.48)	7.90 (21.94)	2.70 (14.16)
Min–max	0–366	0–401	0–106	0–180	0–78	0–341	0–18	0–62	0–93	0–127
Median	0	0	0	0	0	0	0	0	0	0
Acute medications										
Mean (SD)	39.73 (66.61)	19.81 (45.79)	104.8 (202.4)	40.01 (99.05)	34.20 (138.5)	30.56 (91.00)	158.9 (357.3)	45.90 (150.0)	121.9 (216.8)	30.37 (84.75)
Min–max	0–293	0–682	0–772	0–1,079	0–936	0–1,769	0–2,352	0–2,149	0–958	0–925
Median	12	6	9	0	0	0	27	0	37	2
Other medications										
Mean (SD)	40.05 (58.66)	14.53 (31.62)	96.91 (92.40)	25.24 (45.34)	41.58 (98.55)	15.62 (48.46)	160.6 (189.9)	59.59 (111.3)	26.27 (70.70)	9.95 (30.83)
Min–max	0–151	0–151	0–251	0–251	0–370	0–370	0–572	0–572	0–262	0–262
Median	0	0	67	0	0	0	143	0	0	0
Total cost										
Mean (SD)	929.6 (1,605)	216.6 (784.3)	394.9 (526.4)	121.6 (220.1)	373.8 (659.1)	174.0 (509.0)	662.1 (1,298)	207.2 (423.1)	667.5 (1,039)	273.1 (879.3)
Min–max	0–7,233	0–21,042	0–3,417	0–3,047	0–3,894	0–8,629	0–7,433	0–4,523	0–6,059	0–15,684
Median	282	55	251	58	95	19	307	71	230	32

*SD* standard deviation, *n* number of patients

^a^Two patients were excluded because they had extreme values for certain RU parameters (one patient with 45 ER visits and 20 hospitalizations and one patient with 25 X-ray tests)

^b^Includes magnetic resonance imaging, computed tomography, electroencephalogram, and electrocardiogram tests

### Factors influencing total costs

Results of univariate and multivariate models are presented in Table 6. Results of univariate analysis of headache frequency show that CM is associated with higher 3-month total healthcare costs compared to EM across all countries (*P* < 0.05 for all). Total costs for CM and EM by country are also presented in Fig. 1. Overall, the costs of care for EM were highest in Spain followed by the UK, Italy, Germany and France. The costs of CM medical care were highest in Spain followed by the UK and Italy and then France and Germany. After adjusting for headache intensity and comorbidities, CM status was associated with higher total healthcare costs compared to EM for all countries except Germany. Differences were highest in the UK, where CM participants had mean total healthcare costs 3.6-fold higher than EM participants (95 % CI 2.2–6.0) in multivariate analysis, controlling for headache intensity and comorbidities (Table 6). Costs were 2.3-fold higher in France (95 % CI 1.5–3.5), 1.5-fold higher in Germany (95 % CI 0.83–2.7), 2.5-fold higher in Italy (95 % CI 1.5–3.9), and 2.0-fold higher in Spain (95 % CI: 1.2–3.4). Univariate analysis of MIDAS Grades III (moderate disability), IV-A (severe disability), and IV-B (very severe disability) compared to Grade I (little disability) showed that higher disability was associated with higher healthcare costs across all countries (*P* < 0.0001). Headache intensity is generally only associated with higher healthcare-related cost when comparing very severe to mild intensity. The effect of comorbidities varied substantially by country. Presence of pain-related comorbidities were associated with higher cost across all countries, but these associations were only maintained in multivariate analysis for the UK (1.4-fold increase, 95 % CI 1.1–1.7), France (1.5-fold increase, 95 % CI 1.2–1.8), and Germany (1.8-fold increase, 95 % CI 1.5–2.3). Vascular disease risk factors were associated with higher healthcare costs across all countries except Germany, but associations were only maintained in multivariate analysis for the UK (1.2-fold increase, 95 % CI 1.0–1.4) and Spain (1.3-fold increase, 95 % CI 1.0–1.7). Psychiatric comorbidities were associated with higher costs in all countries in univariate analysis (*P* < 0.01 for all comparisons). In multivariate analysis, psychiatric comorbidities were associated with 1.3-fold increase in costs in the UK (95 % CI 1.09–1.5), 1.5-fold increase in Germany (95 % CI 1.2–1.8), and 1.6-fold increase in Italy (95 % CI 1.4–1.9). Statistical significance of psychiatric comorbidities was not maintained in the multivariate models for France or Spain.

**Table 6:** Impact of migraine status on total 3-month health care costs by study country

Determinant	UK (*N* = 1,070)		France (*N* = 1,461)	
	Univariate model	Multivariate model	Univariate model	Multivariate model
	Estimate (CI)^a^	Estimate (CI)^b^	Estimate (CI)^a^	Estimate (CI)^b^
Chronic/episodic migraine				
Chronic vs. episodic migraine	4.4130 (2.8427, 6.8508)	3.6397 (2.2057, 6.0061)	3.1896 (2.1574, 4.7157)	2.3312 (1.5312, 3.5490)
MIDAS				
Mild ($\tilde{6}10$) vs. little disability($\tilde{0}5$)	1.5902 (1.2073, 2.0946)	^c^	1.4431 (1.1851, 1.7571)	^c^
Moderate ($1\tilde{1}20$) vs. little disability ($\tilde{0}5$)	2.3282 (1.7947, 3.0202)	^c^	2.4999 (2.0516, 3.0462)	^c^
Severe ($2\tilde{1}40$) vs. little disability ($\tilde{0}5$)	5.1652 (3.8445, 6.9396)	^c^	4.0463 (3.2386, 5.0555)	^c^
Very severe ($4\tilde{1}270$) vs. little disability ($\tilde{0}5$)	9.3418 (6.6637, 13.0963)	^c^	7.3112 (5.5218, 9.6806)	^c^
Headache intensity				
Moderate ($\tilde{4}6$) vs. mild ($\tilde{0}3$)	0.6117 (0.2920, 1.2811)	0.4130 (0.1805, 0.9450)	1.5814 (0.9769, 2.5602)	1.5795 (0.9426, 2.6469)
Severe ($\tilde{7}9$) vs. mild ($\tilde{0}3$)	1.2451 (0.6164, 2.5148)	0.6758 (0.3068, 1.4886)	3.1136 (1.9559, 4.9566)	2.8558 (1.7326, 4.7070)
Very severe ($\tilde{9}10$) vs. mild ($\tilde{0}3$)	2.4561 (1.2077, 4.9952)	1.1629 (0.5229, 2.5860)	4.5044 (2.7626, 7.3446)	3.4723 (2.0608, 5.8504)
Comorbidities				
Pain-related	1.7386 (1.4500, 2.0847)	1.3719 (1.1285, 1.6678)	1.7395 (1.4450, 2.0941)	1.4746 (1.2285, 1.7700)
Vascular disease risk factors	1.5151 (1.3046, 1.7596)	1.2260 (1.0470, 1.4357)	1.2722 (1.1009, 1.4702)	NS
Vascular disease events	1.4354 (0.9245, 2.2287)	NS	2.3130 (1.4403, 3.7145)	2.3083 (1.3527, 3.9387)
Psychiatric	1.6317 (1.3960, 1.9072)	1.2766 (1.0930, 1.4911)	1.334 (1.1752, 1.5128)	NS
Other	1.2962 (1.1068, 1.5180)	NS	1.4902 (1.3271, 1.6732)	1.2793 (1.1367, 1.4399)

Determinant	Germany (*N* = 1,449)		Italy (*N* = 976)	
	Univariate model	Multivariate model	Univariate model	Multivariate model
	Estimate (CI)^a^	Estimate (CI)^b^	Estimate (CI)^a^	Estimate (CI)^b^
Chronic/episodic migraine				
Chronic vs. episodic migraine	2.1804 (1.2709, 3.7406)	1.4852 (0.8300, 2.6579)	3.3233 (2.0845, 5.2982)	2.4682 (1.5512, 3.9272)
MIDAS				
Mild ($\tilde{6}10$) vs. little disability($\tilde{0}5$)	1.6181 (1.1770, 2.2244)	^c^	1.9129 (1.3876, 2.6371)	^c^
Moderate ($1\tilde{1}20$) vs. little disability ($\tilde{0}5$)	3.1752 (2.3660, 4.2612)	^c^	2.7206 (2.0133, 3.6765)	^c^
Severe ($2\tilde{1}40$) vs. little disability ($\tilde{0}5$)	5.7792 (4.3141, 7.7419)	^c^	4.2869 (3.1435, 5.8462)	^c^
Very severe ($4\tilde{1}270$) vs. little disability ($\tilde{0}5$)	12.4808 (8.8510, 17.5992)	^c^	9.6562 (6.8674, 13.5776)	^c^
Headache intensity				
Moderate ($\tilde{4}6$) vs. mild ($\tilde{0}3$)	4.4639 (2.1656, 9.2016)	3.2341 (1.4718, 7.1068)	0.7072 (0.3917, 1.2768)	0.8813 (0.4872, 1.5942)
Severe ($\tilde{7}9$) vs. mild ($\tilde{0}3$)	4.2579 (2.1435, 8.4581)	3.3449 (1.5570, 7.1858)	1.3401 (0.7719, 2.3264)	1.5133 (0.8667, 2.6421)
Very severe ($\tilde{9}10$) vs. mild ($\tilde{0}3$)	9.8136 (4.8466, 19.8711)	6.3798 (2.9108, 13.9831)	2.1336 (1.1918, 3.8196)	2.1514 (1.1988, 3.8607)
Comorbidities				
Pain-related	2.5335 (2.0185, 3.1799)	1.8498 (1.4605, 2.3429)	1.5156 (1.1373, 2.0197)	NS
Vascular disease risk factors	1.1402 (0.9779, 1.3295)	NS	1.3313 (1.1314, 1.5664)	NS
Vascular disease events	1.8529 (1.2056, 2.8476)	NS	1.6776 (1.0663, 2.6393)	1.5989 (1.0570, 2.4187)
Psychiatric	1.8079 (1.5088, 2.1664)	1.4735 (1.2286, 1.7672)	1.7440 (1.4812, 2.0533)	1.5915 (1.3527, 1.8725)
Other	1.5052 (1.2971, 1.7466)	1.2506 (1.0820, 1.4455)	1.3117 (1.1577, 1.4862)	NS

Determinant	Spain (*N* = 699)			
	Univariate model	Multivariate model		
	Estimate (CI)*	Estimate (CI)		
Chronic/episodic migraine				
Chronic vs. episodic migraine	2.4988 (1.4757, 4.2312)	2.0391 (1.2109, 3.4339)		
MIDAS				
Mild ($\tilde{6}10$) vs. little disability($\tilde{0}5$)	1.7543 (1.1577, 2.6582)	^c^		
Moderate ($1\tilde{1}20$) vs. little disability ($\tilde{0}5$)	3.0981 (2.1461, 4.4724)	^c^		
Severe ($2\tilde{1}40$) vs. little disability ($\tilde{0}5$)	7.0312 (4.4610, 10.6525)	^c^		
Very severe ($4\tilde{1}270$) vs. little disability ($\tilde{0}5$)	9.4031 (5.7907, 15.2691)	^c^		
Headache intensity				
Moderate ($\tilde{4}6$) vs. mild ($\tilde{0}3$)	0.8124 (0.4041, 1.6331)	0.5692 (0.2835, 1.1430)		
Severe ($\tilde{7}9$) vs. mild ($\tilde{0}3$)	0.8638 (0.4588, 1.6265)	0.8742 (0.4612, 1.6571)		
Very severe ($\tilde{9}10$) vs. mild ($\tilde{0}3$)	1.9196 (0.9705, 3.7969)	1.5355 (0.7593, 3.1050)		
Comorbidities				
Pain-related	2.1465 (1.5854, 2.9063)	NS		
Vascular disease risk factors	1.4336 (1.1051, 1.8598)	1.3079 (1.0056, 1.7012)		
Vascular disease events	2.9024 (1.3614, 6.1881)	2.7751 (1.3850, 5.5604)		
Psychiatric	1.3040 (1.0724, 1.5855)	NS		
Other	1.4652 (1.2303, 1.7450)	NS		

Univariate and multivariate models estimated using the gamma log-link function with total 3-month costs as the dependent variable. Estimates are a ratio of the average total costs for patients in a particular migraine, MIDAS, headache intensity, or comorbidity category compared to those in the reference group. For example, total costs for those with chronic migraine in the UK were 4.413 times higher compared to patients with episodic migraine

*CI* 95% confidence interval, *CM* chronic migraine, *EM* episodic migraine, *MIDAS* Migraine Disability Assessment, *NS* not statistically significant (parameter dropped from the model)

^a^Each determinant was assessed separately in univariate regression models that also included age, gender, and education (coefficients not shown)

^b^Determinants assessed jointly in a multivariate regression model including adjustment for age, gender, and education (coefficients not shown)

^c^MIDAS disability was dropped from multivariate models because of correlation with CM/EM status and violation of model assumption of independence between covariates

## Discussion

Using a large international survey of persons with migraine in five European countries, we found that for every country studied, CM is associated with additional health care costs attributable to an increased use of medical services and associate cost. Among participants with CM, the average healthcare costs over 3 months varied greatly, ranging from €373.8 in Germany to €929.6 in the UK. CM was found to be associated with higher total healthcare-related cost even after controlling for headache intensity and comorbidities in all included countries except in Germany. This lack of statistical significance may be attributed to the relatively small difference in average health care costs between CM and EM in Germany (public health care system, controls costs). The difference in mean total healthcare costs between CM and EM per 3 months in Germany was only €199.8 compared to €713.0 in the UK, €273.0 in France, €454.9 in Italy, and €394.4 in Spain. The mean cost of care and the major cost drivers varied widely between countries, potentially reflecting differences in available migraine therapies, delivery of care, cost of services, and structural differences in the healthcare systems of these countries.

Our results suggest that there are differences across the five European countries included in this analysis with respect to migraine management. For example, the percentage of CM participants reporting one or more hospitalizations with overnight stay for migraine was more than twice as high for the UK (8.80 %) compared to any other country (0 % for France, 3.8 % for Germany, 3.6 % for Italy, and 3.6 % for Spain). While these participants accrue higher healthcare costs, the greater proportion of CM participants receiving treatment in the inpatient setting in the UK could be a reflection of better awareness and management of the condition rather than inappropriate care [53]. While a greater proportion of CM participants reported higher use of acute and preventive medications compared to EM in most countries, EM participants had higher rates of acute medication use in the UK and Germany and higher rates of preventive medication use in Italy and Germany. Whether the higher rate of medication use among EM represents a true phenomenon, or whether this finding is an artifact of the survey methodology with potential misclassification of EM and CM remains conjectural. However, it should be noted that less than one-third of CM participants in any country reported use of preventive medications, highlighting that many participants with CM are not receiving therapy which may be beneficial. While uncommon, the proportion of CM participants reporting occipital nerve block procedures were notably higher in the UK compared to other the countries. We feel this may reflect clinical practice, where physicians in the UK are more likely to be trained and actively performing this procedure, reinforcing the external validity of the survey results [54].

No previous studies have compared the cost of CM and EM in Europe. However, the difference in cost between CM and EM was generally similar to that observed in the US and Canadian subgroups of IBMS [18]. Prior studies quantifying the direct costs associated with migraine in general have reported lower estimates than ours, ranging from €12 in the UK to €66 in France, scaled to 2003 prices [55]. The 2004 annual direct cost of migraine was estimated at €127.78 per person in France [56]. In Spain, this was estimated at €198.16 at 2001 prices [57]. Differences in study methodology, and type of healthcare costs included make it difficult, if not impossible to directly compare the results of existing studies. However, older studies likely underestimate the current economic burden of migraine because of the introduction and widespread use of triptan medications for acute management since these studies were conducted. A more recent study on the cost of headache disorders in Europe estimated an annual per-person cost of €1,177 for migraine, with 93 % of this cost attributed to indirect cost (e.g. work absenteeism) [58]. Considering only direct costs thus produces an annual per-person estimate considerably below ours.

This study is subject to a number of limitations and involved several assumptions. First, the majority of cost estimates were derived from publically available sources describing the costs of specific health care resources. Therefore, results are subject to variation in the unit cost estimates that are used as inputs into the economic analysis. Second, resource use data were collected as part of a voluntary online survey where an active e-mail account was a criterion for study. The extent to which restriction to those with internet access limits generalizability to the overall migraine population is unknown. Other limitations include possible selection bias toward more severe migraine participants due to the voluntary nature of the survey. The possibility of selecting a more severely impacted group of migraine participants may explain why our cost estimates are higher than those found in previous studies. The relatively high proportion of participants reporting use of opioids also suggests selection of a highly impacted sample of migraineurs. Healthcare resource use was collected via patient recall over the previous 3-month period (4 weeks for medication use). While recall bias is expected to be minimal for rare events such as emergency room visits and hospitalizations, the self-reported estimates may be less precise for common events such as use of acute medications and physician visits. The diagnostic component but not HRU section of the questionnaire was validated in English [59]. The entire questionnaire was translated and back-translated into other languages, but independent validation studies were not done in each language. Other limitations include the potential for bias in group comparisons due to unmeasurable differences between EM and CM participants, that our sample size for CM was notably smaller than that for EM, and that participants classified as CM were not assessed for meeting the ICHD-II criteria of ≥8 migraine days per month, leaving the potential for misclassification of EM and CM.

CM is associated with greater headache-related disability and impairment of quality of life compared to EM [12–18]. The findings presented here demonstrate that in addition to social and quality of life burden, those with CM also incur greater economic burden. Prophylactic therapies to reduce headache-related disability or therapies that prevent the onset of CM could be important approaches for containing medical costs. The results of this study help to quantify the potential benefit of targeting this highly burdened group of individuals.
